# Maternal characteristics and obstetrical complications impact neonatal outcomes in Indonesia: a prospective study

**DOI:** 10.1186/s12884-017-1280-1

**Published:** 2017-03-28

**Authors:** Trisari Anggondowati, Ayman A. E. El-Mohandes, S. Nurul Qomariyah, Michele Kiely, Judith J. Ryon, Reginald F. Gipson, Benjamin Zinner, Anhari Achadi, Linda L. Wright

**Affiliations:** 1 Center for Family Welfare, Faculty of Public Health, University of Indonesia, Depok, Indonesia; 2 School of Public Health, University of Nebraska Medical Center, Omaha, NE USA; 3 CUNY Graduate School of Public Health and Health Policy, 55 West 125th St., Room 714, New York, NY 10027 USA; 4 (Formerly) John Snow, Inc., Arlington, VA USA; 5 John Snow, Inc., Boston, MA USA; 6 U.S. Agency for International Development, Washington, District of Columbia, USA; 7 School of Public Health, University of Indonesia, Depok, Indonesia; 8 George Washington University School of Medicine and Health Science, Washington, District of Columbia, USA

**Keywords:** Obstetrical, Perinatal death, Asphyxia, Prematurity, Indonesia

## Abstract

**Background:**

We investigated associations between maternal characteristics, access to care, and obstetrical complications including near miss status on admission or during hospitalization on perinatal outcomes among Indonesian singletons.

**Methods:**

We prospectively collected data on inborn singletons at two hospitals in East Java. Data included socio-demographics, reproductive, obstetric and neonatal variables. Reduced multivariable models were constructed. Outcomes of interest included low and very low birthweight (LBW/VLBW), asphyxia and death.

**Results:**

Referral from a care facility was associated with a reduced risk of LBW and VLBW [AOR = 0.28, 95% CI = 0.11–0.69, AOR = 0.18, 95% CI = 0.04–0.75, respectively], stillbirth [AOR = 0.41, 95% CI = 0.18–0.95], and neonatal death [AOR = 0.2, 95% CI = 0.05–0.81]. Mothers age <20 years increased the risk of VLBW [AOR = 6.39, 95% CI = 1.82–22.35] and neonatal death [AOR = 4.10, 95% CI = 1.29–13.02]. Malpresentation on admission increased the risk of asphyxia [AOR = 4.65, 95% CI = 2.23–9.70], stillbirth [AOR = 3.96, 95% CI = 1.41–11.15], and perinatal death [AOR = 3.89 95% CI = 1.42–10.64], as did poor prenatal care (PNC) [AOR = 11.67, 95%CI = 2.71–16.62]. Near-miss on admission increased the risk of neonatal [AOR = 11.67, 95% CI = 2.08–65.65] and perinatal death [AOR = 13.08 95% CI = 3.77–45.37].

**Conclusions:**

Mothers in labor should be encouraged to seek care early and taught to identify early danger signs. Adequate PNC significantly reduced perinatal deaths. Improved hospital management of malpresentation may significantly reduce perinatal morbidity and mortality. The importance of hospital-based prospective studies helps evaluate specific areas of need in training of obstetrical care providers.

**Electronic supplementary material:**

The online version of this article (doi:10.1186/s12884-017-1280-1) contains supplementary material, which is available to authorized users.

## Background

In 2013, 4.6 million infants died worldwide before their first birthday, [1] 50% within the first day and almost 75% within the first week [2]. Another 2.6 million stillbirths occur annually, [3] 25% of which during labor. Most stillbirths and early neonatal deaths are related to complications during birth and could be prevented [4, 5].

The vast majority of perinatal deaths occur in developing countries, including Indonesia, [6] the fourth most populous country in the world [7]. Yet few population-based studies from Indonesia examine the impact of maternal factors on perinatal mortality. Indonesia achieved a significant decline of 24% in the infant mortality rate between 1993 and 1997 (46/1000 live births) and 1998–2002 (35/1000 live births) with only minimal decline since then (34/1000 live births in 2003–2007 and 32/1000 live births in 2008–2012) [8]. This drop was not paralleled by an equal decline in neonatal mortality rate (NMR). Comparison of Indonesia Demographic and Health Surveys (1991, 1994, 1997, 2002–2003, 2007, and 2012) shows NMR constant (22/1000 live births in 1995 and 19/1000 live births in 2005 and 2010) [8]. In association, early NMR was decreasing but has stabilized [8, 9].

The majority of the 6.3 million perinatal deaths occurring annually in developing countries could be avoided if adequate prenatal, intrapartum and neonatal services were available [4, 10, 11]. The lack of investment in improved and accessible hospital services for mothers and infants in Indonesia may be partially responsible for the disproportionately high maternal and associated perinatal mortality rates [12]. In a study examining maternal and neonatal health services in 49 countries, Indonesia received a “weak score” in a rating system for access to maternal health services [13]. We hypothesize that sociodemographic factors, complications of labor, barriers to, and level of care affect maternal as well as neonatal outcomes. A more in-depth understanding of such associations may influence strategic initiatives for training clinical providers and improved hospital facilities.

In this study, we investigated the influence of maternal characteristics and diagnoses, as well as access to hospital care, on birth outcomes among singleton infants born in two district hospitals in East Java.

## Methods

### Study design

This study was a collaboration between the Center for Family Welfare at the School of Public Health at The University of Indonesia and the two district hospitals in East Java. The study was approved by Ethics Clearance Committee of the School of Public Health, University of Indonesia, and the hospitals’ Institutional Review Boards. We obtained verbal consent prior to conducting interviews and chart reviews. Many of the women were not literate. The data collector read the text of the consent to the women (and family). If they agreed to the interview, the interviewer asked for the woman’s signature. If a woman were illiterate, the data collector would note that fact. We established data security measures to ensure the privacy of study participants.

This prospective study occurred in two public district hospitals in East Java Province between October 1, 2009 and March 15, 2010. The first (Hospital A) located in Pasuruan District, is in a coastal area of the Madura Straight. The second district hospital (Hospital B) is located in Kepanjen District, a mountainous region in the south-central part of the province. Hospital A is the primary provider of obstetric care in the district; including obstetric surgery and a large delivery service. Staff include nurses, midwives, obstetricians and pediatricians, but the hospital did not have an adult intensive care unit (ICU). In contrast, the Hospital B has an ICU and is surrounded by 9 private hospitals that also provide obstetric services. Because of its ICU, Hospital B is more likely to receive referrals from other hospitals. Its total delivery caseload is only half that of Hospital A. Neither hospital has a neonatal intensive care unit, which is typical for Indonesian district hospitals.

We collected maternal and neonatal data on obstetric admissions at these hospitals. Data included birth outcomes for all live and stillbirths, socio-demographic characteristics, reproductive history, medical condition(s) on admission, complications during the course of labor, referral (self vs. provider referral) mode of delivery, birth outcomes and condition at discharge.

Near miss events were defined for this study as cases of life-threatening complications in women admitted during pregnancy, labor or postpartum who survived, adapting the criteria originally proposed by Mantel et al. [14] and modified based on input from obstetricians, midwives and epidemiologists [15]. The precise definitions have been previously reported [12].

All mother/infant pairs of singleton hospital births during the study period were eligible if their records were located and could be linked. If the records could not be located or linked, they were excluded, as were readmissions.

Maternal/infant data were linked to investigate the influence of maternal characteristics and medical condition(s) on birth outcomes. Information was linked manually based on the infant’s name and hospital admission number to maternal data using the names of mother and father, as well as the parents’ address, no automated system to link neonatal and maternal records in the hospital was available at that time. Parents’ demographic characteristics, socioeconomic status, access/barriers to care and referral sources were obtained by structured interviews with mothers and accompanying family members during hospitalization. (The interview is available [see Additional file 1.]) Each item was read to the respondents to overcome any literacy issues as a source of bias. Responses were recorded concurrently. We recruited interviewers from the local university who were fluent in the local language and customs in order to improve the quality of the interviews.

### Study population

There were 1240 obstetrical and 910 neonatal admissions to the 2 hospitals during the study period. Nine cases were readmissions, records could not be located for 12 cases (1.3%) and 105 infant admissions were out-born. Of the 784 inborn neonates, 20 sets of twins (*n* = 40) were excluded. Of the 744 remaining eligible singleton live births, 650 (87.4%) were matched with the mother’s record. We were unable to match data for 96 newborn mother dyads. Of the 650, 406 (62.5%) mothers and/or family members were available and consented to participate in an in depth interview to collect socio-demographic and health care utilization data. Women who were available for interview were compared to those not available for interview. The statistical distributions of the two groups were the same for age, gravidity, insurance status, provider vs. self-referral, mode of delivery and severity of illness. When comparing residence, significantly more urban women were interviewed (*p* < 0.001). During the study period 49 stillbirths were reported and were analyzed separately. 28 (57%) of the mothers delivering a stillborn infant consented and participated in the interview.

### Statistical analysis

Bivariate analyses (odds ratios and 95% confidence intervals) were used to evaluate significant associations between risk factors and prospectively selected outcomes (low birthweight (LBW), very low birthweight (VLBW), asphyxia, early neonatal death (<7 days) and perinatal death). Maternal risk factors included maternal reproductive history, socio-demographic characteristics, referral characteristics (transportation, geographical problems, and other administrative barriers to referral), maternal complications (including near-miss and death, maternal medical diagnoses, and mode of delivery). Reduced multivariable models were constructed for each outcome by backwards elimination. The use of reduced multivariable models was intended to generate the most parsimonious model. Variables associated at a significance level of *p* < 0.15 were included in the reduced model. Selection between factors demonstrating strong collinearity (e.g. primigravida status and young maternal age) was based on the relative strength of statistical association, such that the weaker of the two was excluded. We calculated the effect size of the associations in the reduced models using odds ratios and 95% confidence intervals. We used SPSS version 17.0 for Windows for all statistical analyses.

## Results

### Maternal characteristics of those who had live and stillborn infants

The mean age of the mothers was 28 years, with 12% <20 years old and 19% were older than 35, and 48% of deliveries were to primigravidas (See Tables 1 and 2). The national insurance program for the poor, insured more than 50% the women. At the time of the study, women insured under the program for the poor, received care free of charge. Most mothers (79%) lived in a rural environment. The most prevalent admission diagnoses were dystocia (obstructed and prolonged labor) (26.0%), followed by severe preeclampsia/eclampsia (11.3%). Antepartum (APH) and postpartum hemorrhage (PPH) and malpresentation together accounted for another 21.6%. A substantial proportion (41.5%) of admitted patients delivered by cesarean section. Utilizing the classification described by Adisasmita and colleagues, [12, 15] 8.7% of the mothers experienced a near-miss. The medical records documented 73.4% of mothers were referred by another health provider, while 92% of respondents to personal interviews reported either from a single health provider or referral through a sequence of more than one provider (indirect).

**Table 1:** Maternal characteristics of mothers delivering singleton live births and stillbirths at the district study hospitals (October 1, 2009 and March 15, 2010)

Maternal characteristics	Live-birth (*n* = 650)	Stillbirth (*n* = 49)	Total (*n* = 699)	*p* value
Data collected from medical records				
Maternal Age (years)	647 (100)	49 (100)	696 (100)	
< 20	77 (11.9)	7 (14.3)	84 (12.1)	0.294
20–35	430 (69.6)	29 (59.2)	479 (68.8)	
> 35	143 (18.5)	13 (26.5)	133 (19.1)	
Mean ± SD	27.94 ± 7.00	29.65 ± 7.6	28.06 ± 7.05	0.101
Range	14–50	14–44	14–50	
Gravidity	648 (100)	49 (100)	697 (100)	
1	311 (48.0)	19 (38.8)	330 (47.3)	0.460
2–3	246 (38.0)	14 (44.9)	268 (38.5)	
4+	91 (14)	8 (16.3)	99 (14.2)	
Insurance	642 (100)	49 (100)	691 (100)	
Insurance for the poor	350 (54.5)	25 (51.0)	375 (54.3)	0.452
Other insurance	37 (5.8)	5 (10.2)	42 (6.1)	
Out of pocket	255 (39.7)	19 (38.8)	274 (39.7)	
Residence	650 (100)	49 (100)	699 (100)	
Urban	139 (21.4)	10 (20.4)	149 (21.3)	0.872
Rural	511 (78.6)	39 (79.6)	550 (78.7)	
Referral (info from case notes)	650 (100)	49 (100)	699 (100)	
Referred from other health provider	483 (74.3)	30 (61.2)	513 (73.4)	*0.046*
Self referred	167 (25.7)	19 (38.8)	186 (26.6)	
Time of Admission	646 (100)	47 (100)	693 (100)	
Weekdays (07.01–14.00)	273 (42.3)	21 (44.7)	294 (42.4)	0.957
(14.01–21.00)	156 (24.1)	12 (25.5)	168 (24.2)	
(21.01–07.00)	134 (20.7)	9 (19.1)	143 (20.6)	
Weekend	83 (12.9)	5 (10.6)	88 (12.7)	
Time of Delivery	535 (100)	45 (100)	580 (100)	
Weekdays (07.01–14.00)	239 (44.7)	18 (40)	257 (44.3)	0.202
(14.01–21.00)	102 (19.1)	14 (31.1)	116 (20)	
(21.01–07.00)	116 (21.7)	6 (13.3)	122 (21)	
Weekends	78 (14.6)	7 (15.6)	85 (14.7)	
Maternal Diagnosis^a^	650 (100)	49 (100)	699 (100)	
Normal (or minor complications)	75 (11.5)	15 (30.6)	90 (12.9)	*<0.001*
Antepartum hemorrhage	26 (4.0)	4 (8.2)	30 (4.3)	0.166
Postpartum hemorrhage	31 (4.8)	7 (14.3)	38 (5.4)	*0.005*
Severe preeclampsia/eclampsia	72 (11.1)	7 (14.3)	79 (11.3)	0.494
Maternal hypertension not associated with preeclampsia/eclampsia	59 (9.1)	2 (4.1)	61 (8.7)	0.301
PROM	181 (27.8)	4 (8.2)	185 (26.5)	*0.003*
Dystocia	175 (26.9)	6 (12.2)	181 (25.9)	*0.024*
Malpresentation	68 (10.5)	15 (30.6)	83 (11.9)	*<0.001*
Obstetric Infection	9 (1.4)	4 (8.2)	13 (1.9)	*0.001*
Mode of Delivery	650 (100)	49 (100)	699 (100)	
Spontaneous	347 (53.4)	40 (81.6)	387 (55.4)	*0.001*
Assisted vaginal	22 (3.4)	0 (0)	22 (3.1)	
Cesarean	281 (43.2)	9 (18.4)	290 (41.5)	
Severity of illness	650 (100)	49 (100)	699 (100)	
No complications	75 (11.5)	15 (30.6)	90 (12.9)	*<0.001*
Mild-severe complications	521 (80.2)	25 (51.0)	546 (78.1)	
Near-miss or death	54 (8.3)	9 (18.4)	63 (9)	
Near-miss by time	650 (100)	49 (100)	699 (100)	
Non Near-miss	596 (91.7)	42 (85.7)	638 (91.3)	*0.047*
Near-miss at admission	19 (2.9)	5 (10.2)	24 (3.4)	
Near-miss after admission	25 (3.8)	2 (4.1)	27 (3.9)	
Near-miss time unclear	10 (1.5)	0 (0)	10 (1.4)	

Data are mean ± standard deviation or n (%)

^a^Diagnoses are not mutually exclusive

Data in italics have precise significance

**Table 2:** Maternal characteristics for mothers responding to the personal interview (October 1, 2009 and March 15, 2010)

Maternal characteristics	Live birth^a^ (*n* = 406)	Stillbirth^a^ (*n* = 28)	Total (*n* = 434)	*p* value
Women’s education (attended)	406 (100)	28 (100)	434 (100)	
No schooling	6 (1.5)	0 (0)	6 (1.4)	0.548
Elementary	201 (49.5)	10 (35.7)	211 (48.6)	
Junior high	116 (28.6)	11 (39.3)	127 (29.3)	
Senior high	64 (15.8)	6 (21.4)	70 (16.1)	
Academy/University	19 (4.7)	1 (3.6)	20 (4.6)	
Husbands’ education (attended)	392 (100)	27 (100)	419 (100)	
No schooling	7 (1.8)	0 (0)	7 (1.7)	0.652
Elementary	182 (46.4)	14 (51.9)	196 (46.8)	
Junior high	97 (24.7)	6 (22.2)	103 (24.6)	
Senior high	84 (21.4)	7 (25.9)	91 (21.7)	
Academy/University	22 (5.6)	0 (0)	22 (5.3)	
Woman’s occupation	406 (100)	28 (100)	434 (100)	
Not employed	276 (68.0)	16 (57.1)	292 (67.3)	0.237
Employed	130 (32.0)	12 (42.9)	142 (32.7)	
SES	406 (100)	28 (100)	434 (100)	
Quintile 1 (the poorest)	49 (12.1)	3 (10.7)	52 (12)	0.925
Quintile 2	105 (25.9)	8 (28.6)	113 (26)	
Quintile 3	91 (22.4)	6 (21.4)	97 (22.4)	
Quintile 4	95 (23.4)	5 (17.9)	100 (23)	
Quintile 5 (the richest)	66 (16.3)	6 (21.4)	72 (16.6)	
Referral characteristics	406 (100)	28 (100)	434 (100)	
Referred directly to hospital	255 (62.8)	18 (64.3)	273 (62.9)	0.774
Multiple referrals before admission	120 (29.6)	7 (25.0)	127 (29.3)	
Self referred	31 (7.6)	3 (10.7)	34 (7.8)	
Total time travel estimated to reach nearest hospital (hour)	375 (100)	22 (100)	397 (100)	
Mean ± SD	0.63 ± 0.54	0.69 ± 0.70	0.6 ± 0.6	0.590
Range	0.02 – 3.50	0.08 – 3.00	0.02–3.50	
Total time estimated (estimated) by category to nearest hospital	375 (100)	22 (100)	397 (100)	
< 0.5 h	159 (42.4)	10 (45.5)	169 (42.6)	0.450
0.5- < 1 h	112 (29.9)	5 (22.7)	117 (29.5)	
1- < 1.5 h	66 (17.6)	3 (13.6)	69 (17.4)	
1.5- < 2 h	18 (4.8)	3 (13.6)	21 (5.3)	
> =2 h	20 (5.3)	1 (4.5)	21 (5.3)	
Total time since left home until arrived at study hospital (hour)	329 (100)	22 (100)	351 (100)	
Mean ± SD	12.3 ± 20.2	6.2 ± 10.4	11.9 ± 19.8	0.162
Range	0.28 – 159.8	0.7 – 48.0	0.28 – 159.8	
Total time traveled to reach study hospital	329 (100)	22 (100)	351 (100)	
< 0.5 h	8 (2.4)	0 (0)	8 (2.3)	0.254
0.5- < 1 h	13 (4.0)	3 (13.6)	16 (4.6)	
1- < 1.5 h	34 (10.3)	3 (13.6)	37 (10.5)	
1.5- < 2 h	28 (8.5)	2 (9.1)	22 (6.3)	
> =2 h	246 (74.8)	14 (63.6)	268 (76.4)	
Type of transportation used to reach study hospital (actual)	367 (100)	26 (100)	393 (100)	
By foot/ *Becak*/bicycle	11 (3.0)	0 (0)	11 (2.8)	0.174
Motorbike/*ojek*	55 (15.0)	8 (30.8)	63 (16)	
Ambulance	128 (34.9)	5 (19.2)	133 (33.8)	
Public transportation	121 (33.0)	9 (34.6)	130 (33.1)	
Private car (non ambulance)	52 (14.2)	4 (15.4)	56 (14.2)	
Distance (km) to reach nearest hospital (estimated)	185 (100)	14 (100)	199 (100)	
Mean ± SD	12.9 ± 15.7	17.2 ± 19.8	13.2 ± 16	0.334
Range	0.05–80	0.05–60	0.05–80	
Distance to reach nearest hospital (estimated)	185 (100)	14 (100)	199 (100)	
< 5 km	64 (34.6)	4 (28.6)	68 (34.2)	0.773
5–9.9 km	59 (31.9)	4 (28.6)	63 (31.7)	
10+ km	62 (33.5)	6 (42.9)	68 (34.2)	
Distance (km) traveled to reach study hospital (actual)	131 (100)	10 (100)	141 (100)	
Mean ± SD	20.0 ± 18.4	32.5 ± 23.3	20.8 ± 19.0	0.056
Range	1 - 85	11 – 83	1 - 85	
Distance to reach study hospital (actual) – categorized	131 (100)	10 (100)	141 (100)	
< 5 km	34 (26.0)	1 (10.0)	35 (24.8)	0.116
5–9.9 km	22 (16.8)	0 (0)	22 (15.6)	
10+ km	75 (57.3)	9 (90.0)	84 (59.6)	
Reported barrier to referral	406 (100)	28 (100)	434 (100)	
Personal barrier	26 (6.4)	0 (0)	26 (6)	0.167
Transportation barrier	54 (13.3)	3 (10.7)	57 (13.1)	0.695
Geographic barrier	71 (17.5)	10 (35.7)	81 (18.7)	*0.017*
Fund barrier	65 (16)	6 (21.4)	71 (16.4)	0.453
Administrative barrier	38 (9.4)	5 (17.9)	43 (9.9)	0.145
Prenatal Care	406 (100)	28 (100)	434 (100)	
Yes	395 (97.3)	26 (92.9)	421 (97)	0.183
Number of visits 4+	325 (83.8)	17 (63.0)	342 (82.4)	*0.006*
Initiated 1st trimester	319 (83.5)	25 (89.3)	344 (83.9)	0.612
Initiated 2nd trimester	54 (14.1)	3 (10.7)	57 (13.9)	
Initiated 3rd trimester	9 (2.4)	0 (0)	9 (2.2)	

^a^Data are mean ± standard deviation or n(%)

Data in italics have precise significance

Responses from the patient interviews (Table 2) showed that 48.6% of the mothers and almost half of the fathers (46.8%) had only an elementary school education, 32.7% of the mothers reported that they were employed and 38.0% belonged to the two poorest quintiles of the socioeconomic classification. Most of the mothers (72%) lived less than one hour away from the nearest hospital, 52% used either motorbike or public transportation or walked to get to the hospital. Only 33.8% used an ambulance. 31.8% of the mothers reported transportation or geographic barriers had interfered access to hospital care. Over 25% reported financial and administrative barriers to receiving care. Despite the reported barriers to hospital care, 97% had received prenatal care, which is mostly available at the village level. 93.3% reported at least 4 visits, and 82.3% of mothers had initiated care in the first trimester.

### Maternal risk factors associated with stillbirth

On admission, 84% of 49 stillborn infants had no fetal heart rate upon arrival at the hospital. Mothers delivering a stillborn were significantly different from those delivering a singleton live birth (Tables 1 and 2), including a higher percentage of PPH (*p* = 0.005), dystocia (*p* = 0.024), malpresentation (*p* <0.001) and delivery via C-section (*p* < 0.001), to have been self-referred (*p* = .046) and to report geographic barriers as interfering with access to care (*p* = 0.017). They were more likely to be classified as near-miss on admission or during hospitalization (*p* = 0.047). Mothers delivering a stillbirth were also less likely to have received the recommended ≥4 prenatal care visits (*p* < 0.001). Stillborn infants weighed significantly less than live born infants (*p* < .0001) with 48.5% weighing less than 1500 grams (Data not shown).

### Neonatal characteristics

Of the 650 live born infants studied, there were 34 neonatal deaths, with only one occurring after the first week of life. Early NMR amongst this group of singleton live born infants was 52.3/1000 live births (See Table 3). Of the singleton live born infants, 3.4% weighed <1500 grams (VLBW) and 15.2% weighed < 2500 grams (LBW). The most commonly reported neonatal diagnosis was asphyxia (15.8%). This was corroborated by 12.6% of infants with 5-minute Apgar scores <5.

**Table 3:** Neonatal characteristics of singleton infants born at the two district hospitals (October 1, 2009 and March 15, 2010)

Neonatal characteristics	*n* = 650 (%)
Infant Sex	
Male	358 (55.1)
Female	292 (44.9)
Birthweight (kg)	
< 1000	9 (1.4)
1000–1499	13 (2)
1500–1999	19 (2.9)
2000–2499	58 (8.9)
2500–2999	200 (30.8)
3000–3999	330 (50.8)
≥ 4000	16 (2.5)
Not recorded	5 (0.8)
Apgar Score at 5 min	
< 5	82 (12.6)
≥ 5	565 (86.9)
Not recorded	3 (0.5)
Breastfed	
Yes	317 (48.8)
Not recorded	107 (16.5)
Neonatal Diagnoses^a^	
Normal	242 (39.3)
LBW	99 (15.2)
VLBW	22 (3.4)
Respiratory distress	38 (5.8)
Asphyxia	103 (15.8)
Sepsis^b^	10 (1.5)
Meconium stained amniotic fluid	33 (5.1)
At risk for sepsis^b^	63 (9.7)
Post-Caesarean Section	43 (6.6)
Neonatal death	34 (5.2)
No diagnosis recorded in medical record	38 (5.8)

No blood cultures done, diagnosis based on symptoms and associated with documented risk

^a^Diagnoses are not mutually exclusive

^b^At risk for sepsis neonate asymptomatic but associated with one of the following: maternal fever, prolonged rupture of membranes

### Maternal risk factors associated with Low birthweight, very low birthweight, neonatal asphyxia, stillbirth, perinatal death and neonatal death

In the reduced logistic model, referral from another health care facility was associated with a reduced risk of LBW [AOR = 0.28, 95% CI = 0.11, 0.69, [VLBW [AOR = 0.18, 95% CI = 0.04, 0.75], stillbirth [AOR = 0.41, 95% CI = 0.18, 0.95], and neonatal death [AOR = 0.20, 95% CI = 0.05, 0.81] (See Table 4).

**Table 4:** Maternal risk factors associated with singleton newborn outcomes (October 1, 2009 and March 15, 2010)

Maternal risk factor	Bivariate Analysis Odds Ratios [95% CI]	*p*-value	Reduced Logistic Model Adjusted Odds Ratios [95% CI]	*p*-value
Low Birthweight (*n* = 357)				
Young maternal age (<20 years)	*1.83 [1.03, 3.25]*	*0.04*		
Primagravida	*1.78 [1.52, 2.74]*	*0.01*		
Rural residence	1.81 [0.99, 3.29]	0.05		
Referred from another facility	*0.47 [0.24, 1.90]*	*0.02*	*﻿0.28 [0.11, 0.69]﻿*	*0.01*
Antepartum hemorrhage	*2.57 [1.09, 6.08]*	*0.03*		
Eclampsia	1.68 [0.92, 3.07]	0.09		
Near miss	*2.08 [1.08, 3.98]*	*0.03*		
Time to reach nearest hospital >1 h	1.88 [0.88, 4.02]	0.10		
Prenatal care visits (<4 visits)	*2.66 [1.06, 6.70]*	*0.04*		
Very Low Birthweight (*n* = 388)				
Young maternal age (<20 years)	*3.72 [1.47, 9.44]*	*<0.01*	*6.39 [1.82, 22.35]*	*<0.01*
Referral from another facility	*0.22 [0.08, 0.58]*	*<0.01*	*0.18 [0.04, 0.75]*	*0.02*
Mother presented with complication	*0.33 [0.13, 0.87]*	*0.03*		
Antepartum hemorrhage	*4.15 [1.15, 15.04]*	*0.03*		
Prenatal care visits (<4 visits)	3.27 [0.67, 15.98]	0.14		
Asphyxia (*n* = 357)				
Primagravida	1.42 [0.93, 2.17]	0.11		
Insurance for the poor	1.54 [0.99, 2.34]	0.05		
Rural residence	*4.36 [1.98, 9.63]*	*<0.001*	*5.37 [1.98, 18.16]*	*<0.01*
Near miss after admission	2.14 [0.88, 5.27]	0.10		
Malpresentation	*3.47 [2.00, 6.03]*	*<0.001*	*4.65 [2.23, 9.70]*	*<0.001*
Low education	2.10 [0.96, 4.58]	0.06		
Multiple referrals	0.57 [0.30, 1.08]	0.09		
Prenatal care visits (<4 visits)	1.98 [0.79, 4.90]	0.14		
Stillbirth (*n* = 699)				
Referral from another facility	*0.40 [0.18, 0.86]*	*0.019*	*0.41 (0.18, 0.95]*	*0.37*
Postpartum hemorrhage	*3.33 [1.38, 8.01]*	*0.007*		
PROM	*0.23 [0.08, 0.65]*	*0.006*	*0.27 [0.09, 0.76]*	*0.014*
Malpresentation	*3.78 [1.96, 7.29]*	*<0.001*	*4.27 [2.11, 8.62]*	*<0.001*
Dystocia	*0.38 [0.16, 0.91]*	*0.029*		
Caesarean Section	*0.30 [0.14, 0.62]*	*0.001*	*0.28 [0.13, 0.60]*	*0.001*
Near miss on admission	*3.77 [1.35, 10.59]*	*0.012*		
Near miss at any time	*2.48 [1.14, 5.39]*	*0.021*	*3.54 [1.53, 8.21]*	*0.003*
Neonatal deaths (*n* = 375)				
Young maternal age (<20 years)	*2.90 [1.30, 6.48]*	*<0.01*	*4.10 [1.29, 13.02]*	*0.02*
Advanced maternal age	0.26 [0.06, 1.12]	0.07		
Primagravida	2.06 [1.00, 4.24]	0.05		
Referral from another facility	*0.31 [0.13, 0.75]*	*0.01*	*0.20 [0.05, 0.81]*	*0.02*
Antepartum hemorrhage	2.5 [0.71, 8.76]	0.15		
Postpartum hemorrhage	2.9 [0.96, 8.85]	0.06	*4.11 [1.03, 16.39]*	*0.05*
Near miss on admission	*2.58 [1.07, 6.22]*	*0.03*	*11.67 [2.08, 65.65]*	*<0.01*
Caesarean Section	0.53 [0.25, 1.13]	0.10		
Near miss at any time	*5.42 [2.44, 12.04]*	*<0.001*	*7.5 [2.24, 25.08]*	*<0.01*
Belonging to the two poorest income quintiles	0.36 [0.10, 1.30]	0.12		
Time to reach nearest hospital >1 h	0.76 [0.17, 1.31]	0.15		
Perinatal deaths (*n* = 348)				
Young maternal age 9 < 20 years)	1.99 [1.09, 3.61]	0.02		
Referral from another facility	*0.33 [0.18, 0.61]*	*<0.001*		
Time of delivery (at night)	0.68 [0.41, 1.11]	0.12		
Antepartum hemorrhage	2.19 [0.92, 5.15]	0.08		
Postpartum hemorrhage	*2.70 [1.32, 5.52]*	*<0.01*	*3.96 [1.41, 11.15]*	*<0.01*
Malpresentation	*2.59 [1.47, 4.57]*	*<0.01*	*3.89 [1.42, 10.64]*	*<0.01*
Near miss on admission	*5.27 [2.42, 11.47]*	*<0.001*	*13.08 [3.77, 45.37]*	<0.001
Near miss at any time	*3.24 [1.81, 5.77]*	*<0.001*	*6.00 [2.32, 15.50]*	*<0.001*
Mother presented with complication	*0.41 [0.23, 0.71]*	*<0.01*	*0.30 [0.11, 0.81]*	*0.02*
Caesarean Section	*0.37 [0.22, 0.64]*	*<0.001*		
Prenatal care visits (<4 visits)	*5.17 [2.34, 11.39]*	*<0.001*	*6.70 [2.71, 16.62]*	*<0.001*

Data in italics have precise significance

Young maternal age significantly increased the risk for VLBW [AOR = 6.39, 95% CI = 1.82, 22.35] and neonatal death [AOR = 4.10, 95% CI = 1.29, 13.02]. Maternal factors significantly associated with neonatal asphyxia included rural residence [AOR = 5.37, 95% CI = 1.59, 18.16] and malpresentation during delivery [AOR = 4.65, 95% CI = 2.23, 9.70]. Prolonged rupture of membranes and delivery by C-section was associated with a reduced risk of stillbirth [AOR = 0.28, 95% CI = 0.11, 0.69; AOR = 0.28, 95% CI = 0.13, 0.60, respectively). Factors associated with a significantly increased risk of stillbirth included malpresentation [AOR = 3.96, 95% CI = 1.41, 11.15] and near miss at any time [AOR = 3.54, 95% CI = 1.53, 8.21]. Factors associated with a significantly increased risk of perinatal death included PPH [AOR = 3.96, 95% CI = 1.41, 11.15], malpresentation [AOR = 3.89, 95% CI = 1.42, 10.64] and near miss on admission [AOR = 13.08, 95% CI = 3.77, 45.37], near miss at any time, [AOR = 6.00, 95% CI = 2.32, 15.50], and <4 prenatal care visits [AOR = 6.7, 95% CI = 2.71, 16.62].

## Discussion

This study is one of only three in the literature examining the impact of maternal diagnoses on infant outcomes using hospital data collected prospectively. The first was published in 1991 in India [16]. Another recent study in the West Bank and Gaza Strip was based on prospective data collected at the household level [17]. Our study is the only one in the literature using prospectively collected data that has linked specific maternal characteristics and diagnoses to the incidence of neonatal asphyxia as an outcome. Another study, using the 2002–2003 Indonesia Demographic and Health Survey, reported that “other complications” significantly increased the risk of neonatal mortality [18]. Previous studies have examined perinatal mortality as outcomes and used perinatal asphyxia as a risk factor for death.

Our study shows strong evidence of high perinatal and early neonatal mortality and morbidity among infants born to women delivering in two district hospitals in Indonesia. The early NMR measured at these two hospitals was 52.3/1000 live births, 2.75 times the nationally reported NMR of 19/1000 [8, 19]. A reduction of hospital-based NMR may significantly impact national neonatal survival. Malpresentation was identified in this study as associated a 4-fold increased risk for stillbirth, which is in agreement with previous findings in Ethiopia, [20] the West Bank, [17] and India [16]. A stronger emphases on early diagnosis, referral, and proper obstetrical management of malpresentation/dystocia may have reduced mortality rates significantly in this population. Other complications such as preeclampsia/eclampsia seemed to be managed more effectively in Indonesia since they were not associated with increased perinatal death unlike the findings from other developing countries [16, 17, 20]. C-section as a protective factor could have been confounded by the fact that C-section was only performed when the fetus had a greater likelihood of being born alive. Prolonged rupture of membranes retained the least significant protective effect for stillbirth, possibly due to the longer time period after presenting to the healthcare delivery system, allowing for intervention.

The preliminary analyses in this study show a constellation of maternal factors associated with poor birth outcomes, including young maternal age, living in rural settings, distance from hospital, poverty, lower education and unemployment. These findings confirm those reported by others [16, 17, 20]. Rural residence or living a distance from the hospital creates problems with access to care, be it due to time, transportation or other geographic issues. These risk factors are not unique to Indonesia, since living in rural settings, [21–23] being younger, [24–26] poor, [22, 27, 28] less educated, [24, 29, 30] and unemployed^1^ [21, 31, 32] have all been shown to raise a woman’s risk for poor birth outcomes in developed and developing countries. On the other hand, most of these risk factors lost their significance in reduced models that took into consideration specific obstetrical complications and severity of maternal illness. This is a unique contribution of our study. The findings on young maternal age, as a predominant risk factor for VLBW and neonatal death, highlight the need to support family planning services to young prospective parents. Although there has been an increase in the age of first marriage, the median age is only 20.4 years. Among women who have completed a secondary education, the median age at first marriage is 22.9 years compared with 17.2 years among women who have no education [8]. Creating specialized programs for newly married couples and young pregnant women in rural districts would target the population at highest risk. These couples would benefit from enhanced knowledge on safe pregnancy, prenatal care, delivery plans, early danger signs during pregnancy and labor, emergency readiness, and the importance of secondary and tertiary levels of care in ensuring optimal outcomes if complications occur.

Mothers referred to the hospital by another health care provider, presumably at a lower level of care had significantly improved outcomes. The investments Indonesia has made in training at the primary health care level is reflected in that finding, as well as the protective effect of adequate prenatal care generally provided at the community level. Referral during labor from a health facility has a protective effect that self-referral does not and highlights the importance of early engagement of mothers with the health care delivery system. It also emphasizes the need to upgrade the education of primary health care providers in appropriate and timely referrals to protect mothers from arriving at the hospital with irreversible medical complication(s). A measure of the need to improve the referral system is that 29% of women in the study reported multiple referral points to reach the hospital. Despite geographical barriers and distance from the health care facility, seeking care or advice from a primary care provider or a lower level of care reduced the risk of stillbirth and VLBW in this population. The latter finding may suggest adequate management of preterm labor in primary care facilities that are able to provide conservative interventions such as hydration and bed rest.

Half of mothers presenting for obstetric care at these hospitals were primigravida; these mothers presented with a statistically lower risk for LBW. It is possible that families prioritized healthcare to first time pregnant women more than among women during subsequent pregnancies.

Mothers presenting to the hospitals had minimal education, and relied on the national insurance program for the poor to support the cost of their hospitalization. Eighty percent of them lived in a rural environment and described transportation and geographical barriers as the most important obstacles to hospitalization. Rural women were significantly more likely to go through a series of multiple referrals until they reached the hospital. In the absence of reliable transportation, precious time was wasted. This could have contributed to the association between rural residence and a 5-fold increase in the risk for neonatal asphyxia, also significantly associated with malpresentation. The possibility of better training for delivery techniques of a malpresenting fetus at lower levels of care could have contributed significantly in reducing the risk of asphyxia.

In 2007, Indonesia launched their birth preparedness and complication readiness program (P4K) that outlines multiple levels of involvement: the woman, her family, the community, the health facility, the provider and the policymaker. The goal is that women reach professional delivery care when labor begins and reduce delays that occur when mothers, in labor, experience obstetric complications. Ensuring implementation of such programs to include other family members and important community leaders may enhance the effectiveness of such programs.

A limitation of this study was that its reliance on the quality of hospital records, partially mitigated by corroborating data through multiple sources. Data for socio-economic and access to care variables were obtained from patient interviews, which may be subject to recall bias. Interviewing patients and family during hospitalization presents challenges in obtaining accurate information. Strengths of the study include that the data abstractors and interviewers were trained clinicians recruited locally. There was consistent crosschecking between registers, medical records and patient interviews to ensure the quality of the data.

## Conclusions

Our results emphasize the need for improving the awareness of timely and appropriate referral of mothers diagnosed with conditions frequently associated with poor neonatal outcomes. Our conclusions highlight the interconnectedness and complex relationships between personal, ecological and health care factors involved in perinatal risk. There is no question that a well-designed regional perinatal care network with well-trained providers is essential to maximize good neonatal outcomes; [33] reducing economic barriers to care can only improve outcomes to the extent allowable by the quality of care provided. Access to prenatal care as well as secondary and tertiary levels of care, in addition to readily available transport systems can predictably reduce adverse outcomes both for the mother [12] and the infant. Maternal risk factors such as young maternal age will improve with time and are best changed by education, behavioral interventions and changes in underlying socio-economic factors. Unpredicted and undiagnosed complications could be mitigated by accurate and early diagnosis during pregnancy and during labor with appropriate and timely referral.

This study presents a strong case for the need for systems planning. The evidence presented here indicates that improvements can be made on many fronts, from encouraging mothers to delay childbearing into their twenties, to elimination of barriers to prenatal care, prompt referral and quality hospital care once a woman is admitted to the hospital. The potential savings, particularly in the prevention of perinatal morbidity and mortality, may be substantial.

## Additional file

Additional file 1: QMP5. English version of maternal interview, including demographic data, care/referral data, social and medical support, problems, obstetric history, prenatal care, delivery history, and brief medical history (chronic and infectious disease(s)). (PDF 170 kb)

## Abbreviations

AOR: Adjusted odds ratio
APH: Antepartum hemorrhage
ICU: Intensive care unit
LBW: Low birth weight
NMR: Neonatal mortality rate
PNC: Prenatal care
PPH: Postpartum hemorrhage
VLBW: Very low birth weight

## Footnotes

1 Unemployment here implies lack of access to money and does not address issues related to physically difficult or environmentally dangerous jobs.

## References

[1] WHO | infant mortality. 2015. http://www.who.int/gho/child_health/mortality/neonatal_infant_text/en/. Accessed 13 May 2015.

[2] UNICEF. Newborn health. 2015. https://www.unicef.org/health/newbornhealth.html. Accessed 22 May 2017.

[3] Cousens S, Blencowe H, Stanton C (2011). National, regional, and worldwide estimates of stillbirth rates in 2009 with trends since 1995: A systematic analysis. Lancet.

[4] Weiner R, Ronsmans C, Dorman E, Jilo H, Muhoro A, Shulman C (2003). Labour complications remain the most important risk factors for perinatal mortality in rural kenya. Bull World Health Organ.

[5] Metaferia AM, Muula AS. Stillbirths and hospital early neonatal deaths at queen elizabeth central hospital, blantyre-malawi. Int Arch Med. 2009;2(1):25-7682-2-25. 10.1186/1755-7682-2-25 PMC2746193 19719841

[6] Anonymous (2006). Neonatal and perinatal mortality: Country, regional and global estimates.

[7] Hogan MC, Foreman KJ, Naghavi M (2010). Maternal mortality for 181 countries, 1980-2008: A systematic analysis of progress towards millennium development goal 5. Lancet.

[8] Indonesia demographic and health survey 2012 - FR275.pdf. 2015. http://dhsprogram.com/pubs/pdf/FR275/FR275.pdf. Accessed 15 May 2015.

[9] Hatt L, Stanton C, Ronsmans C, Makowiecka K, Adisasmita A (2009). Did professional attendance at home births improve early neonatal survival in indonesia?. Health Policy Plan.

[10] De Brouwere V, Richard F, Witter S (2010). Access to maternal and perinatal health services: Lessons from successful and less successful examples of improving access to safe delivery and care of the newborn. Trop Med Int Health.

[11] Ngoc NT, Merialdi M, Abdel-Aleem H (2006). Causes of stillbirths and early neonatal deaths: Data from 7993 pregnancies in six developing countries. Bull World Health Organ.

[12] Adisasmita A, Smith CV, El-Mohandes AA (2015). Maternal characteristics and clinical diagnoses influence obstetrical outcomes in indonesia. Matern Child Health J.

[13] Bulatao RA, Ross JA (2003). Which health services reduce maternal mortality? evidence from ratings of maternal health services. Trop Med Int Health.

[14] Mantel GD, Buchmann E, Rees H, Pattinson RC (1998). Severe acute maternal morbidity: A pilot study of a definition for a near-miss. Br J Obstet Gynaecol.

[15] Adisasmita A, Deviany PE, Nandiaty F, Stanton C, Ronsmans C. Obstetric near miss and deaths in public and private hospitals in indonesia. BMC Pregnancy Childbirth. 2008;8:10-2393-8-10. 10.1186/1471-2393-8-10 PMC2311270 18366625

[16] Mavalankar DV, Trivedi CR, Gray RH (1991). Levels and risk factors for perinatal mortality in ahmedabad, india. Bull World Health Organ.

[17] Kalter HD, Khazen RR, Barghouthi M, Odeh M (2008). Prospective community-based cluster census and case-control study of stillbirths and neonatal deaths in the west bank and gaza strip. Paediatr Perinat Epidemiol.

[18] Titaley CR, Dibley MJ, Agho K, Roberts CL, Hall J. Determinants of neonatal mortality in indonesia. BMC Public Health. 2008;8:232-2458-8-232. 10.1186/1471-2458-8-232 PMC2478684 18613953

[19] Statistics Indonesia (Badan Pusat Statistik—BPS) and Macro International (2008). Indonesia demographic and health survey 2007.

[20] Bayou G, Berhan Y (2012). Perinatal mortality and associated risk factors: A case control study. Ethiop J Health Sci.

[21] Sparks PJ, McLaughlin DK, Stokes CS (2009). Differential neonatal and postneonatal infant mortality rates across US counties: The role of socioeconomic conditions and rurality. J Rural Health.

[22] Yao N, Matthews SA, Hillemeier MM (2012). White infant mortality in appalachian states, 1976-1980 and 1996-2000: Changing patterns and persistent disparities. J Rural Health.

[23] Van de Poel E, O’Donnell O, Van Doorslaer E (2009). What explains the rural-urban gap in infant mortality: Household or community characteristics?. Demography.

[24] Partridge S, Balayla J, Holcroft CA, Abenhaim HA (2012). Inadequate prenatal care utilization and risks of infant mortality and poor birth outcome: A retrospective analysis of 28,729,765 U.S. deliveries over 8 years. Am J Perinatol.

[25] Chen XK, Wen SW, Fleming N, Demissie K, Rhoads GG, Walker M (2007). Teenage pregnancy and adverse birth outcomes: A large population based retrospective cohort study. Int J Epidemiol.

[26] Ali M, Lulseged S (1997). Factors influencing adolescent birth outcome. Ethiop Med J.

[27] Kramer MS, Seguin L, Lydon J, Goulet L (2000). Socio-economic disparities in pregnancy outcome: Why do the poor fare so poorly?. Paediatr Perinat Epidemiol.

[28] Blumenshine P, Egerter S, Barclay CJ, Cubbin C, Braveman PA (2010). Socioeconomic disparities in adverse birth outcomes: A systematic review. Am J Prev Med.

[29] Meyer JD, Warren N, Reisine S (2010). Racial and ethnic disparities in low birth weight delivery associated with maternal occupational characteristics. Am J Ind Med.

[30] Pamuk ER, Fuchs R, Lutz W (2011). Comparing relative effects of education and economic resources on infant mortality in developing countries. Popul Dev Rev.

[31] Scharber H (2014). Does “out of work” get into the womb? exploring the relationship between unemployment and adverse birth outcomes. J Health Soc Behav.

[32] Arafa MA, Amine T, Abdel Fattah M (2007). Association of maternal work with adverse perinatal outcome. Can J Public Health.

[33] Achadi EL, Achadi A, Pambudi E, Marzoeki P. A study on the implementation of the jampersal policy in indonesia < br />. 2015. http://www-wds.worldbank.org/external/default/WDSContentServer/WDSP/IB/2014/10/13/000333037_20141013140335/Rendered/PDF/913250WP0UHC0C00Box385331B00PUBLIC0.pdf. Updated 2014. Accessed 14 July 2015.

