# Supplementary material for: Maternal characteristics and obstetrical complications impact neonatal outcomes in Indonesia: a prospective study
Source: BMC Pregnancy Childbirth. 2017 Mar 28;17:100. doi: 10.1186/s12884-017-1280-1 (PMC5371232; doi:10.1186/s12884-017-1280-1)
Supplement: Additional file 1: — QMP5. English version of maternal interview, including demographic data, care/referral data, social and medical support, problems, obstetric history, prenatal care, delivery history, and brief medical history (chronic and infectious disease(s)). (PDF 170 kb) [file 12884_2017_1280_MOESM1_ESM.pdf]

Study No.: I__I / I__I / I__I / I__I / I__I__I__I
Hos DC R/P O/N Case No.

QMP5

QUality and **A**ccess to **PE**rinatal hospital **C**are at the district level (**QUAPEC**)

INFORMED CONSENT – MATERNAL
For obstetric patient or her family who is most knowledgeable about the patient's condition and characteristics

1. Study number
2. Name of the woman ___________________
3. Case note number

RESPONDENT INFORMED CONSENT:

SEEK RESPONDENT'S AGREEMENT TO PARTICIPATE BY READING THE STATEMENT BELOW.

My name is ___________. I am from the University of Indonesia. Under official permission from the hospital, we are conducting a study to explore information from the patients, mainly about referral and patient's characteristics. The purpose of this activity is mainly to find out any difficulties faced by patients in obtaining health care. I would like to interview you about (the condition of [full name of the patient] or about your condition –depends on whether respondent is the patient her self or other family member*) ___________. The information you give us will be treated in the strictest confidence. Participation in this interview is entirely voluntary. You are free not to take part at all or to stop the interview at any time and there will be no implication on the quality and cost of care provided to you by this hospital. However, your answers are very important to us and will help to improve the delivery of health services and access in the utilization of health services, especially for mothers and babies in Indonesia. The interview will last approximately 30 minutes. Are you willing to be interviewed? .... If Yes, would you please sign this form to indicate your consent.*

Full name of respondent ________________________ Signature ________________________

1. Agree to be interviewed
2. Do not agree

(circle one of the above accordingly)

Do you have any questions about the interview or may I begin the interview?
If the informant is not willing to be interviewed, thank him/her and end the interview.

Hospital Code	1. Malang Hospital; 2. Pasuruan Hospital		
Date of admission	DD/MM/YYYY	__ __ / __ __ / __ __ __ __	
Time of interview	HH:MM	Start __ __ : __ __	End __ __ : __ __
Result of interview	1. Completed<br>2. Incomplete, reason ......<br>(e.g: patient stopped the interview)<br>3. Not done, reason......<br>(e.g.: patient refused, discharged)		

Information on form management

	Filling in form	Check 1 (data collector)	Check 2 (field supervisor)	Entry 1	Entry 2
Name					
Code					
Date	__ / __ / ______	__ / __ / ______	__ / __ / ______	__ / __ / ______	__ / __ / ______
Signature					

NOT FOR DISTRIBUTION

QMP5. Form in INPATIENT/FAMILY INTERVIEW
For ALL Obstetric Admission

No.	Questions	Response Categories	Code
**I. RESPONDENT'S IDENTITY**			
1.1	Name of respondent		
1.2	Sex of respondent	1. Male 2. Female	☐
1.3	Relationship of informant to the patient	1. Husband<br>2. Parent<br>3. Parent in law<br>4. Sibling<br>5. Sibling in law<br>6. Other relative<br>7. Respondent is the patient<br>8. Other, specify	☐
**II. CHARACTERISTICS OF THE WOMAN AND HER FAMILY**			
2.1	Name of the patient		
2.2	Age of the patient [NAME]	__________ years	☐☐
2.3	Occupation of the patient		
2.4	What is the highest level of school [NAME] attended?	0=No schooling<br>1=primary<br>2=junior high<br>3=senior high<br>4=academy<br>5=university ☐<br>6=other, specify ________	☐
2.5	What is the highest (grade/year) [NAME] completed at that level?	Year/grade .... 88=Don't know	☐☐
2.6	Name of husband		
2.7	Age of husband	_________ years 88=Don't know	☐☐
2.8	Husband's occupation		
2.9	What is the highest level of school that the husband attended?	0=No schooling<br>1=primary<br>2=junior high<br>3=senior high<br>4=academy<br>5=university ☐<br>6=other, specify _______<br>8=don't know	☐
2.10	What is the highest (grade/year) that the husband completed at that level?	Year/grade .... 88=Don't know	☐☐
2.11	Does anyone else in your household have higher level of education than you or your husband?	1=Yes 0=No 8=Don't know	☐☐
	If Q2.11 is YES, ask Q2.12 and 2.13 for member of household referred in Q2.11. If NO, skip to Q2.14.		

NOT FOR DISTRIBUTION

2.12	What is the highest level of school that he/she (person you referred to in Q2.11) attended?	0=No schooling<br>1=primary<br>2=junior high<br>3=senior high<br>4=academy<br>5=university<br>6=other, specify _______<br>7=NA<br>8=don't know	
2.13	What is the highest (grade/year) that he/she completed at that level?	Year/grade ....<br>77=NA 88=Don't know	
2.14	Address	Village (Desa/Kelurahan) : ______________________________<br>RT/RW : _____ / _____<br>Sub-district : ______________________________<br>District (Kabupaten/Kodya): ______________________________	
2.15	Distance from home to nearest hospital	_________ km 8888=Don't know	
2.16	The distance above (2.15) can be reached in ...... using what kind of mode of transportation?	_______ minutes 8888=Don't know<br><br>Mode of transportation:<br>1. By foot<br>2. Motorbike/*ojek*<br>3. Ambulance<br>4. Public transportation/rent<br>5. Private car<br>6. Other (specify)	
2.17	Distance from home to PHC	_________ km 8888=Don't know	
2.18	The distance above (2.17) can be reached in ...... using what kind of mode of transportation?	_______ minutes 8888=Don't know<br><br>Mode of transportation:<br>1. By foot<br>2. Motorbike/*ojek*<br>3. Ambulance<br>4. Public transportation/rent<br>5. Private car<br>6. Other (specify)	
2.19	Distance from home to midwife's residence	_________ km 8888=Don't know	
2.20	The distance above (2.19) can be reached in ...... using what kind of mode of transportation?	_______ minutes 8888=Don't know<br><br>Mode of transportation:<br>1. By foot<br>2. Motorbike/*ojek*<br>3. Ambulance<br>4. Public transportation/rent<br>5. Private car<br>6. Other (specify)	
2.21	Number of person live in your household		
2.22	Other member of the family who are chronically ill and dependent		
2.22.1	Number of family member who are chronically ill and dependent		
2.22.2	Specify the disease:	Person 1<br>Person 2<br>Person 3<br>Person 4	

NOT FOR DISTRIBUTION

Would you please tell me about the care she received prior her admission in this hospital

III. REFERRAL INFORMATION					
**Information**	**Place/Person First**	**Place/Person Second**	**Place/Person Third**	**Place/Person Fourth**	**Place/Person Fifth**
a. Place/person contacted/sought for help					
b. Was this a public or a private sector provider?<br>1. Public 2. Private 8. DK					
c. Who accompanied the patient? (ANSWERS COULD BE MORE THAN 1)<br>1. Husband 2. Parent<br>3. Parent in Law 4. Sibling<br>5. Other, specify 8. DK					
d. When help/care was sought?	Date: ________<br>Time: _____	Date: ________<br>Time: _____	Date: ________<br>Time: _____	Date: ________<br>Time: _____	Date: ________<br>Time: _____
e. Where help/care was provided?					
f. Distance to referral place (answer for both km and minute), from previous place	_______ kms<br>_______ minutes	_______ kms<br>_______ minutes	_______ kms<br>_______ minutes	_______ kms<br>_______ minutes	_______ kms<br>_______ minutes
g. Mode of transportation<br>1. By foot<br>2. Motorbike/*ojek*<br>3. Ambulance<br>4. Public transportation/rent<br>5. Private car<br>6. Other (specify)					
h. When did the provider reach the mother's place, or mother reach the provider's place?	Date: ________<br>Time: _____	Date: ________<br>Time: _____	Date: ________<br>Time: _____	Date: ________<br>Time: _____	Date: ________<br>Time: _____
i. How long did it take for the mother to receive the help/care since she reached the provider?	_________ hours<br>_________ minute	_________ hours<br>_________ minute	_________ hours<br>_________ minute	_________ hours<br>_________ minute	_________ hours<br>_________ minute
j. If there was any delay in receiving					

NOT FOR DISTRIBUTION

**III. REFERRAL INFORMATION**					
**Information**	**Place/Person**<br>**First**	**Place/Person**<br>**Second**	**Place/Person**<br>**Third**	**Place/Person**<br>**Fourth**	**Place/Person**<br>**Fifth**
care, what was the reason?					
k. Care provided: (record all types of care given including drug administration etc.)					
l. Summary of conditions					

a. If the mother was referred to more than 5 providers or places, who and where were the referrals made to? ____________________

b. Please provide the description of each referral event, and any problem occurred during individual referral process (e.g. problems with transportation, lack of drug supplies, etc).

____
____
____
____
____
____
____
____
____
____

NOT FOR DISTRIBUTION

Please describe any supports or assistance received by the woman in relation to her index pregnancy or current illness

IV. SUPPORT FROM THE COMMUNITY OR OTHERS			
	**What was the form of support?**	**Who gave the support?**<br>**Ask ”who else”**	**When was it (e.g. prior admission to the hospital, during the woman's pregnancy)**
4.1	Money		
4.2	Transportation		
4.3	Blood		
4.4	Attaining an *SKTM* (a letter stated that the woman is from a poor family)		
4.5	Other, specify (e.g. assistance in taking care of the woman's children): ………..		
4.6	Have you or anyone in your household participated in community health activities organized by … (read all responses). Circle all that apply	COMMUNITY HEALTH COMMITTEE A<br>POSYANDU B<br>BIDAN DI DESA / POLINDES C<br>PKK D<br>PUSKESMAS E<br>OTHER (SPECIFY________________) F	
4.7	Is there any P4K sticker sticked on the wall/door of [NAM]		

NOT FOR DISTRIBUTION

Were any of these problems below present?

V. PROBLEMS FOUND				
	**System**	**Example**	**Code**	**Description**
5.1	Personal/Family/ Community	Delay in mother seeking help/care<br>1=Yes 2=No 8=Unknown	☐	
5.2		Refusal of treatment or admission<br>1=Yes 2=No 8=Unknown	☐	
5.3	Logistical systems: Transportation, Communication, Access (Distance, Culture, Socio-economy))	Lack of transportation from home to health facility<br>1=Yes 2=No 8=Unknown	☐	
5.4		Lack of transportation between health care facilities<br>1=Yes 2=No 8=Unknown	☐	
5.5		Geographical problem to reach health care facility<br>1=Yes 2=No 8=Unknown	☐	
5.6		Lack of fund for referral and delivery care<br>1=Yes 2=No 8=Unknown	☐	
5.7	Administration	Lack of access to Letter of Poverty Proof (SKTM)<br>1=Yes 2=No 8=Unknown	☐	
5.8	Is there any P4K sticker sticked on the wall/door	1=Yes 2=No 8=Unknown	☐	

**No.**	**Questions**	**Response Categories**	**Code**
	**VI. PREVIOUS OBSTETRIC HISTORY**		
6.1	Age at first pregnancy of the patient	_____ year	☐☐
6.2	Number of previous births of the patient	1. Live-birth: _________<br>2. Still-birth: __________<br>3. Abortion, including ectopic pregnancy, Hydatidiform mole:_________<br>99. Unknown /No information	1. ☐☐<br>2. ☐☐<br>3. ☐☐
6.3	Had any of the patient's child died	1. Yes 2. No<br>9. Unknown /No information	☐
6.4	If Q6.3 is YES, please list age of death of the patient's child	(see table below)	
6.5	Number of current living children		☐☐
6.6	Number of children born premature	1. Yes, spcify ....<br>2. No<br>8. Unknown /No information	☐<br>☐☐
6.7	Number of children born less than 2500 gram	1. Yes, specify ....<br>2. No<br>8. Unknown /No information	☐<br>☐☐
6.8	Interval between previous and index pregnancy	_______ months<br>888. Unknown/No information	☐☐
6.9	Where did the patient deliver her previous pregnancy?	1. Home<br>2. Midwife's home<br>3. Health centre	☐

6.4 table:

No.	Age	Year/month/days	No.	Age	Year/month/days

NOT FOR DISTRIBUTION

		4. Clinic<br>5. Hospital<br>6. Others, specify ________________<br>7. NA	____________________
**History of complication during previous pregnancy / delivery**			
6.10	Ante-partum hemorrhage	1. Yes 2. No 8. NA 9. Unknown	☐
6.11	Massive bleeding after delivery (post-partum)	1. Yes 2. No 8. NA 9. Unknown	☐
6.12	Retained placenta / retained product	1. Yes 2. No 8. NA 9. Unknown	☐
6.13	Obstructed labor / abnormal lie (mal-presentation)	1. Yes 2. No 8. NA 9. Unknown	☐
6.14	Preeclampsia (Severe headache / blurred vision / high blood pressure)	1. Yes 2. No 8. NA 9. Unknown	☐
6.15	Seizure due to eclampsia	1. Yes 2. No 8. NA 9. Unknown	☐
6.16	Caesarean section	1. Yes 2. No 8. NA 9. Unknown	☐
6.17	Was the baby large?	1. Yes 2. No 8. NA 9. Unknown	☐
6.18	Did the mother have an infection or a fever?	1. Yes 2. No 8. NA 9. Unknown	
6.19	Other, specify	1. Yes 2. No 8. NA 9. Unknown	☐<br>____________________

No.	Questions	Response Categories	Code
**VII. PRE-NATAL CARE FOR THE INDEX PREGNANCY**			
7.1	Did the mother have any antenatal care during this pregnancy?	1. Yes<br>2. No<br>9. Unknown	☐
7.2	If yes, where?<br>CHECK ALL THAT APPLY	1. Yes 2. No 7. NA 8. Don't know	
		PUBLIC:	
		Hospital	☐
		Community health center	☐
		Clinic	☐
		Maternity clinic	☐
		PRIVATE:	
		Hospital	☐
		Clinic	☐
		Maternity clinic	☐
		Private doctor	☐
		Private midwife	☐
		Private midwife 'delima'	☐
		COMMUNITY:	
		Maternity hut/polindes	☐
		Posyandu	☐
		In other home/TBA home	☐
		OTHER, specify	☐

NOT FOR DISTRIBUTION

7.3	Antenatal care provider	1. Obstetrician<br>2. General practitioner<br>3. Midwife<br>4. Nurse<br>8. NA	
7.4	Number of ANC visits	1. Trimester I ______ visits<br>2. Trimester II ______ visits<br>3. Trimester III ______ visits<br>Total: _________ visits<br>88. NA	
7.5	When did the mother had her first antenatal care	.... week of pregnancy	
7.6	Did the mother see a TBA during your last pregnancy?	1. Yes, specify service received:<br>_________________________<br>0. No	________________________

No.	Questions	Response Categories	Code
		**VIII. DELIVERY HISTORY**	
8.1	Did the mother come for post abortion care?	1. Yes 2. No 7. NA 9. Unknown	
Fill in 8.2 – 8.12 with NA for condition: abortion, ectopic pregnancy, mola, hiperemesis gravidarum, and other condition when no delivery			
8.2	Date of delivery	___/___/___ dd/mm/yy<br>___/___/___ dd/mm/yy<br>___/___/___ dd/mm/yy<br>77/77/77=NA	Fill in the boxes for each baby<br>/ /<br>/ /<br>/ /
8.3	Time of delivery	___: ___ hour/mnt<br>77. NA<br>88. Unknown/No information	Fill in the boxes for each baby<br>Baby1 -<br>Baby 2 -<br>Baby 3 -
8.4	How long did the mother have cramps (adequate and consistent) until delivering the baby?	______minute<br>77. NA<br>88. Unknown/No information	
8.5	During labour, which part of the baby came out first?	1. Head<br>2. Leg<br>3. Breech<br>4. Arm<br>8. Unknown	Fill in the boxes for each baby<br>Baby1<br>Baby2<br>Baby3
8.6	Mode of delivery	1. Spontaneous/Normal<br>2. Vacuum /forcep extraction<br>3. Breech manouver<br>4. External version<br>5. Embryotomy/decapitation/evisceration<br>6. C-section/per abdominam	Fill in the box for each baby<br>Baby1<br>Baby2<br>Baby3
8.7	Place of delivery	01. Own home/ family's home<br>02. TBA's home<br>03. Birthing hut (Polindes)<br>04. Auxiliary PHC (Pustu)<br>05. Rumah bidan<br>06. Hospital<br>07. On the way to health facility<br>08. Other: ________________	Fill in the box for each baby<br>Baby1<br>Baby2<br>Baby3
8.8	Birth attendant	01. TBA	Fill in the box for each baby

NOT FOR DISTRIBUTION

		02. Family / relative<br>03. Midwife – public (bidan-di-desa)<br>04. Midwife – private (non bidan-di-desa)<br>05. Midwife - unspecified<br>06. General practitioner – public<br>07. General practitioner - private<br>08. Obstetrician – public<br>09. Obstetrician – private<br>10. Other, specify ___________<br>77. Tujuh percent	Baby1 ☐<br>Baby2 ☐<br>Baby3 ☐
8.9	If the mother died, how long did the mother have cramps before she died?	______minute<br>7777. NA (not in labour yet or mother died before delivery)<br>8888. Unknown/No information	☐☐☐☐
8.10	Was there any massive bleeding during delivery?	1. Yes 2. No<br>7. NA 8. Unknown	☐
8.11	If yes, when	1. Yes 2. No 7. NA 8. Unknown<br>a. Before labor<br>b. During labor<br>c. After delivery<br>d. Abortion	☐<br>☐<br>☐<br>☐
8.12	Was the placenta delivered?	1. Yes 2. No<br>7. NA 8. Unknown	☐

IX. HISTORY OF ANY CHRONIC AND INFECTIOUS DISEASE (should be extracted from MR)					
9.1	Bronchiale asthma	1. Yes	2. No	8. Dknow	☐
9.2	Epilepsy	1. Yes	2. No	8. Dknow	☐
9.3	Chronic Hypertension before pregnancy	1. Yes	2. No	8. Dknow	☐
9.4	Heart disease	1. Yes	2. No	8. Dknow	☐
9.5	Diabetes before pregnancy	1. Yes	2. No	8. Dknow	☐
9.6	Chronic kidney disease	1. Yes	2. No	8. Dknow	☐
9.7	Malaria	1. Yes	2. No	8. Dknow	☐
9.8	Tuberculosis	1. Yes	2. No	8. Dknow	☐
9.9	Other, specify	1. Yes	2. No	8. Dknow	☐

**No.**	**Questions**	**Response Categories**	**Code**
		**X. ECONOMIC STATUS**	
10.1	House ownership	1. Own house<br>2. Family's house<br>3. Rent<br>4. Official house<br>5. Other, specify	☐
10.2	Do you or does your household have any of the following?<br><br>READ OUT THE RESPONSES	1=Yes 2=No 8=Don't know<br>A. Electricity<br>B. Radio/transistor<br>C. Television<br>D. Telephone/handphone<br>E. Refrigerator<br>F. Bicycle<br>G. Motorcycle<br>H. Car<br>I. Truck	☐<br>☐<br>☐<br>☐<br>☐<br>☐<br>☐<br>☐<br>☐

NOT FOR DISTRIBUTION

10.3	Floor	1. Natural floor (earth/sand/mud)<br>2. Wood<br>3. Bamboo<br>4. Brick/concrete/cement<br>5. Ceramic<br>6. Tile<br>7. Other, specify, ___________________<br>8. Don't know	☐
10.4	Wall	1. Bamboo<br>2. Wood<br>3. Brick/concrete<br>4. Semi-permanent<br>5. Other, specify, ___________________<br>8. Don't know	☐
10.5	Are you a member of any of the following health insurance / benefit programs?	1. Yes 2. No 8. Don't know<br>1. Out-of-pocket payment<br>2. Private insurance<br>3. Askes PNS/paid by company/Jamsostek<br>4. Askes Gakin/Jamkesmas/Askeskin/ Jamkesda/ SKTM<br>5. Other, specify__________________	☐<br>☐<br>☐<br>☐<br>☐<br>☐

No.	Questions	Response Categories	Code
		**XI. COST OF CARE**	
11.1	How much is the total cost spent by the family for [NAME]'s care in this hospital	***Total cost of care, includes for buying medicines from outside the hospital; excluded cost for the family's transportation and accommodation.***	☐☐☐☐☐☐☐☐
11.2	Method of payment and share of total cost for this care	1. Yes 2. No 8. Don't know<br>1. Out-of-pocket payment<br>2. Private insurance<br>3. Askes PNS/paid by company/Jamsostek<br>4. Askes Gakin/Jamkesmas/Askeskin/ Jamkesda/ SKTM<br>5. Other, specify__________________	☐<br>☐<br>☐<br>☐<br>☐<br>☐

CHECK THE COMPLETENESS OF QUESTIONNAIRE.

IF ALL HAS BEEN COMPLETED, WRITE DOWN TIME OF THE INTERVIEW END AND THANK THE RESPONDENT.

NOT FOR DISTRIBUTION
